# Elucidating Biological Functions of 9-*cis*-Epoxycarotenoid Dioxygenase Genes Involved in Seed Dormancy in *Paeonia lactiflora*

**DOI:** 10.3390/plants12040710

**Published:** 2023-02-06

**Authors:** Riwen Fei, Shixin Guan, Siyang Duan, Jiayuan Ge, Tianyi Sun, Xiaomei Sun

**Affiliations:** 1 College of Horticulture, Shenyang Agricultural University, Shenyang 110866, China; 2 Key Laboratory of Forest Tree Genetics Breeding and Cultivation of Liaoning Province, College of Forestry, Shenyang Agricultural University, Shenyang 110866, China; 3 College of Forestry, Shenyang Agricultural University, Shenyang 110866, China

**Keywords:** *Paeonia lactiflora* pall., abscisic acid, *NCEDs*, seed dormancy, germination

## Abstract

Abscisic acid (ABA) is a major phytohormone affecting seed dormancy and germination in plants. ABA is synthesized mainly through the C40 carotenoid pathway. In the ABA biosynthesis pathway, 9-*cis*-epoxycarotenoid dioxygenase (NCED) is a key rate-limiting enzyme that regulates the accumulation and content of ABA. However, the role of the *NCED* gene in perennial plants with complex seed dormancy remains largely unknown. Here, we cloned two differentially expressed paralogs of herbaceous peony *NCED* genes, named *PlNCED1* and *PlNCED2*, and further identified their involvement in seed dormancy from perennial herbaceous peony experiencing complex double seed dormancy. The deduced PlNCED amino acid sequences had high sequence homology with *NCED* sequences from other plants and contained the typical conserved RPE65 domain of the NCED family. Phylogenetic analysis showed that *PlNCED1* and *PlNCED2* have a close relationship with *PoNCED* in *Paeonia ostii* and *VvNCED6* in *Vitis vinifera*, respectively. A subcellular localization assay demonstrated that the *PlNCED1* protein resided within the nucleus, while the *PlNCED2* protein was located in the cytoplasm, indicating their different roles in the biosynthesis of ABA. Furthermore, the content of endogenous ABA in transgenic calluses showed that *PlNCEDs* were positively correlated with ABA content. Both *PlNCED* transgenic *Arabidopsis* lines and the functional complementation of *Arabidopsis NCED* mutants found that *PlNCEDs* promoted seed dormancy and delayed seed germination. These results reveal that *PlNCEDs* participate in the seed dormancy of herbaceous peony by regulating the accumulation of endogenous ABA.

## 1. Introduction

Phytohormones are important regulators of seed dormancy, among which ABA plays a major role [1,2,3]. Studies have shown that ABA-deficient mutants of *A. thaliana*, tomato, and maize undergo early dormancy breaking and enter the germination stage, whereas plants overexpressing the ABA biosynthetic enzyme show prolonged dormancy [4,5,6,7]. Studies also demonstrated that the change in endogenous ABA content significantly positively correlates with the degree of seed dormancy [8,9].

In plants, it is known that several phytohormones are involved in seed dormancy and germination [10,11,12]. Among them, endogenous ABA content is significantly positively correlated with herbaceous peony seed dormancy, while low ABA content promotes seed germination [13]. The final concentration of endogenous ABA in plant seeds depends on the dynamic balance of ABA synthesis and catabolism [1,14]. *NCED* and *CYP707A* are two key enzymes in ABA anabolic and catabolic pathways, respectively. Studies show that NCED has a common role in regulating ABA synthesis and seed dormancy in plants. For example, *A. thaliana* contains five *NCED* family members, of which *AtNCED5* is up-regulated at the late stage of seed maturation and cooperates with *AtNCED6* and *AtNCED9* to enhance seed dormancy by controlling ABA levels [15,16]. *LeNCED1* transgenic tomato plants enhanced ABA biosynthesis and increased seed dormancy [17]. The variation trend of ABA content was consistent with that of *AhNCED1* gene transcription in peanut [18]. *PtNCED1* directly regulated orchid seed dormancy and was involved in ABA content [19]. In addition, *NCED* can also affect other physiological functions of plants by changing endogenous ABA content. Overexpressing *PvNCED1* enhanced drought tolerance by manipulating ABA levels in tobacco [4]. Silencing *AcNCED1* blocked ABA biosynthesis and delayed kiwifruit softening [20]. *PpNCED1* and *PpNCED5* can cooperatively control ABA biosynthesis and affect fruit ripening and senescence in peach fruit [21]. However, not all *NCED* family members regulate ABA synthesis. For example, a total of 23 *NCED* genes were identified in cotton. Among them, only the expression of *GhNCED5*, *GhNCED6*, and *GhNCED13* was similar to the change in ABA content, which could play a role in ABA biosynthesis [22].

Herbaceous peony (*Paeonia lactiflora* Pall.) is an herbaceous perennial flower of *Paeoniaceae*. In the long-term systematic evolutionary process, herbaceous peony seeds evolved a unique double dormancy characteristic of the epicotyl and hypocotyl [23]. In the breeding process, seed dormancy is often not released or incompletely released, which greatly reduces the germination rate and seriously affects cultivation and production [24]. Thus, understanding the mechanisms associated with herbaceous peony seed dormancy is beneficial to greatly promote the breeding of new varieties. At present, research on the seed dormancy release technology for herbaceous peony mainly focuses on mechanical scarification, low temperature, and exogenous hormone treatment [25,26,27]. However, the knowledge of the molecular mechanisms underlying the seed dormancy of herbaceous peony is relatively limited. Using previously published transcriptomes from herbaceous peony seeds pre- and post-double dormancy release [28], here, we were able to identify candidate genes that were associated with double dormancy in herbaceous peony. Specifically, we identified ten family members of *PlNCEDs* involved in ABA biosynthesis. Among them, *PlNCED1* (c53147_g1) and *PlNCED2* (c69372_g1) showed significantly differential expression. We subsequently cloned and performed expressional analysis, subcellular localization analysis, and functional characterization of *PlNCED1* and *PlNCED2* in *Paeonia lactiflora*. Our results demonstrate that the genes encoding NCED1 and NCED2 regulate ABA synthesis and consequentially affect the herbaceous peony seed dormancy process.

## 2. Materials and Methods

### 2.1. Plant Material and Growth Condition

**Figure 1:**
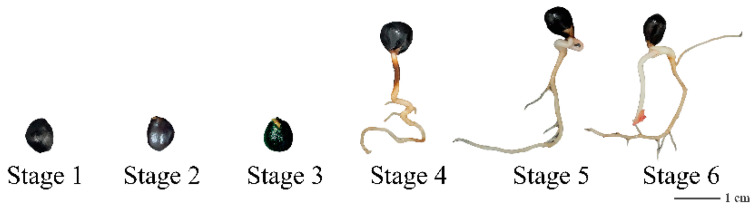
Seeds of herbaceous peony at six different dormancy release stages.

Herbaceous peony hybrid seeds (‘Fen Yu Nu’ × ‘Fen Yu Lou’) were harvested in the Shenyang Agricultural University germplasm resources nursery (Shenyang, Liaoning, China) in August 2019. Filled hybrid seeds were used for variable temperature stratification using a previous method [29]. According to the observation of the seed anatomical structure [29], seeds in six key dormancy release stages were collected: stage 1 (S1: dry seed), stage 2 (S2: imbibition seed), stage 3 (S3: the radicle breaking of seed coat), stage 4 (S4: the length of the seed root is 3–4 cm), stage 5 (S5: the basal part of the seed root turns red), and stage 6 (S6: seed germ breakout) (Figure 1). The cotyledons used as explants were obtained using the conventional embryo induction method [30]. Then, the explants were transferred to an MS callus induction and proliferation medium containing 0.5 mg/L 2,4-dichlorophenoxyacetic acid, 0.5 mg/L α-naphthalene acetic acid, 0.5 mg/L thidiazuron, and 1 g/L polyvinyl pyrrolidone (PVP). The *nced5-2* (GK_328D05) and *nced9-1* (SALK_033388) genes, which are in the Col-0 background, were obtained from the Arabidopsis Biological Resource Center (ABRC, http://abrc.osu.edu). Homozygous mutants were screened and validated by PCR using the left and right genomic primers (LP and RP) and the T-DNA left border primer (LB) (Supplementary Table S1). Seeds of *A. thaliana* WT (Col-0) and mutants were grown following previously reported methods [31].

### 2.2. RNA Extraction, cDNA Synthesis, and qRT-PCR

Total RNA was extracted using the RNAprep pure Plant Kit (TianGen, Beijing, China). cDNA was synthesized using the PrimeScript™ RT Master Mix kit (Perfect Real Time) (Takara, Dalian, China). Based on the full-length coding sequences (CDSs) of *PlNCEDs* in transcriptome data, qRT-PCR primers were designed with the Primer Premier 5.0 software. *PlACTIN* (GenBank accession number JN105299.1) was used as the reference gene [32]. The primers used for qRT-PCR are listed in Supplementary Table S1. qRT-PCR was performed using TB Green^®^ Premix Ex Taq™ II (Tli RNaseH Plus) (Takara, Dalian, China). The reactions were accomplished according to the two-step method—holding stage: 95 °C for 30 s; cycling stage: 40 cycles of 95 °C for 5 s, 60 °C for 30 s; and melt curve stage: 95 °C for 15 s, 60 °C for 1 min, 95 °C for 15 s. Each experiment was performed with three biological and three technical replicates. The relative expression levels of genes were calculated according to the 2^−ΔΔCt^ method, and the error bars represent the standard error from three independent experiments. The results were analyzed by GraphPad Prism 8.0 for ANOVA.

### 2.3. Cloning and Sequence Analysis

We obtained the CDSs of *PlNCEDs* from transcriptome data. The amino acid sequences of *PlNCEDs* were deduced using ORF Finder (https://www.ncbi.nlm.nih.gov/orffinder/) (accessed on 8 December 2022). A phylogenetic tree was constructed using MEGA 7.0 software with the neighbor-joining method, applying bootstrap analysis with 1000 replicates, and iTOL v6 (https://itol.embl.de/) (accessed on 13 January 2023) was used to optimize the trees. The conserved domains were predicted by National Center for Biotechnology Information (NCBI) online software (https://www.ncbi.nlm.nih.gov/Structure/cdd/wrpsb.cgi) (accessed on 13 January 2023). The protein physicochemical properties were analyzed using the Expasy ProtParam tool (http://web.expasy.org/protparam/) (accessed on 8 December 2022).

Using total RNA as the template, 1st-strand cDNA was synthesized using 3’ RACE adaptor primers. According to the CDSs of *PlNCEDs*, we designed the specific outer and inner primers (Supplementary Table S1) to amplify the 3’ untranslated region (UTR) sequences of *PlNCEDs* using 3’-Full RACE Core Set with PrimeScript™ RTase Kit (Takara, Dalian, China). The miRNA binding sites of 3’ UTR sequences were predicted using MiRanda software.

Genomic DNA was extracted using the Plant Genome DNA Rapid Extraction Kit (Aidlab, Beijing, China). According to the known verified intronless sequences, three specific primers were designed, namely, SP1, SP2, and SP3 (Supplementary Table S1), to amplify the 5’ end sequences of *PlNCEDs* containing 5’ UTR and promoter regions using the Genome Walking Kit (Takara, Dalian, China). *Cis*-acting elements of the promoter were analyzed using PlantCARE.

### 2.4. Subcellular Localization Analysis

*Arabidopsis thaliana* leaf protoplasts were extracted using the *Arabidopsis* Protoplast Preparation and Transformation Kit (Coolaber, Beijing, China) for subcellular localization. The CDSs of *PlNCEDs* were cloned into the 16318-hGFP vector and fused in-frame with the hGFP sequence under the control of the CaMV 35S promoter. The 16318-hGFP empty vector was used as a blank control. After 16 h of incubation in darkness, the green fluorescence protein (GFP) fluorescence was captured by an ultra-high-resolution laser scan confocal microscope (Leica TCS SP8 STED, Wetzlar, Germany).

### 2.5. Functional Analysis

To silence *PlNCED* expression in the herbaceous peony callus, the fragments of *PlNCEDs* (*PlNCED1*: 565 bp; *PlNCED2*: 387 bp) were each amplified and recombined into the linearized pTRV2 empty vector. The CDSs of *PlNCEDs* were separately inserted downstream of 35S in the pCAMBIA1300-35S-flag vector. The pTRV2-PlNCED and 35S::PlNCED vectors were transformed into EHA105-competent cells. *Agrobacterium* containing pTRV1 and *Agrobacterium* containing pTRV2-PlNCEDs were mixed in a 1:1 volume ratio for the preparation of the callus infection solution. The overexpression and silencing experiment in the herbaceous peony callus was performed according to our previous infection method [33]. After the resistance screening of the culture, part of the PlNCED transgenic callus was taken for qRT-PCR identification. The extraction, purification, and determination of the endogenous levels of ABA in the positive transgenic callus of *PlNCEDs* using an enzyme-linked immunosorbent assay (ELISA) were performed as described by He [34].

We obtained two *A. thaliana* homozygous T-DNA mutants (Figure S1): *nced5-2* and *nced9-1*. *NCED* mutants have interrupted 9-*cis*-epoxycarotenoid dioxygenase genes that result in plants that are deficient in the plant growth regulator abscisic acid. To make the transgenic line and functional complementation line of *A. thaliana*, the 35S::PlNCED vectors were transformed into *Agrobacterium* strain GV3101 and then used to infect inflorescences of *A. thaliana* WT (Col-0) and homozygous mutants (*nced5-2* and *nced9-1*) using the floral-dip method [35], respectively. The transgenic line and functional complementation line were screened on ½ MS medium plates that contained 50 mg/L kanamycin. The seed germination rate was measured in WT, homozygous mutants, functional complementation lines, and transgenic lines (stable T_3_-generation genetic lines) of *A. thaliana*, which were grown at the same time under 16 h light and 8 h dark conditions at 22 °C.

## 3. Results

### 3.1. Cloning and Sequence Analysis of PlNCEDs

**Figure 2:**
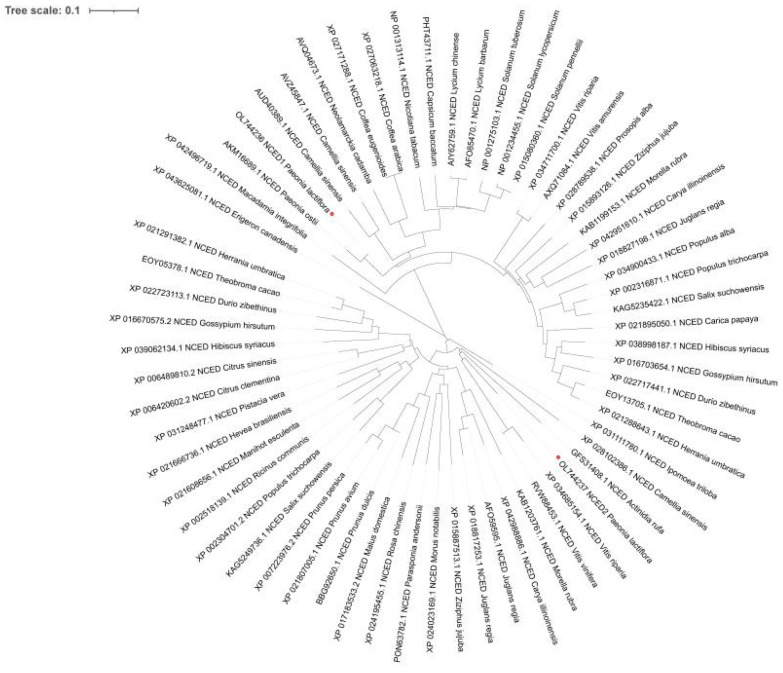
Neighbor-joining phylogenetic tree of *PlNCEDs*. PlNCED1 and PlNCED2 are indicated with red dots. A neighbor-joining tree was constructed based on amino acid sequences of NCED protein from different plants using MEGA 7.0 software. The bootstrap values of the branches were obtained by testing the tree 1000 times.

The full-length cDNA sequences of *PlNCEDs* were isolated and deposited in GenBank (GenBank accession numbers—*PlNCED1*: OL744236; *PlNCED2*: OL744237). *PlNCED1* and *PlNCED2* contained 1518 bp and 1326 bp open reading frames, encoding 505 and 441 amino acids, respectively (Figure S2). NCBI tblastx results displayed their homology to the *NCED* genes of other plant species. PoNCED from *Paeonia ostii* (74.70%) and JrNCED from *Juglans regia* (62.43%) had the highest identity with PlNCED1 and PlNCED2, respectively (Supplementary Tables S2 and S3). This similarity demonstrated that PlNCEDs are relatively conserved among diverse plant species. PlNCEDs had the typical conserved RPE65 domain of the NCED family (Figure S3), which is related to the degradation of carotenoids in plants. Phylogenetic analysis results indicated that PlNCED1 and PlNCED2 are closely related to the NCED proteins in *Paeonia ostii*, *Vitis riparia*, and *Vitis vinifera*, respectively (Figure 2). In addition, based on DNA sequences, we identified putative microRNA (miRNA) and transcription factor binding sites in the 3’UTR and promoter regions of *PlNCEDs* (Supplementary Tables S4–S7). Among the miRNAs that may target the 3’UTRs of *PlNCEDs* genes, six miRNAs, namely, miR837-5p, miR5640, miR319c, miR6425a/b/c/d/e-5p, miR168a, and miR5304, are related to plant development (Supplementary Tables S4 and S5). Among the *cis*-acting elements that may bind to the promoter sequences of *PlNCED* genes, six cis-acting elements, namely, TCA-element, ABRE, AuxRR-core, TGA-box, TGACG-motif, and CGTCA-motif, are related to phytohormone responses (Supplementary Tables S6 and S7). These results suggest that *PlNCEDs* may be involved in plant development mediated by ABA and other phytohormones.

### 3.2. Expression Analysis of PlNCEDs

To explore the correlation between the expression of *PlNCED* genes and ABA accumulation in herbaceous peony seeds, we performed qRT-PCR using seeds at stages 1-6. Our results indicated that the expression levels and trends of *PlNCED1* and *PlNCED2* dynamically varied during seed dormancy release (Figure S4). The expression level of *PlNCED1* increased sharply from stage 1 to stage 2 and then decreased significantly at stage 3, where seeds experienced hypocotyl dormancy release (Figure S4). After the completion of the hypocotyl dormancy release process, the expression level of *PlNCED1* increased incrementally from stage 4 to stage 6 (Figure S4). Conversely, we observed different trajectories for the expression of the *PlNCED2* gene. Overall, *PlNCED2* maintained a high level of expression at stages 1-2, and it displayed a decreasing trend from stages 2 to 6 (Figure S4). Correspondingly, the ABA content in each of the six seed dormancy release stages largely declined from S1 to S6 (Figure S5) [36]. By comparing the dynamics of *PlNCED* expression and ABA content during the process of dormancy release, we demonstrated that only *PlNCED2* expression was positively associated with ABA accumulation.

### 3.3. Subcellular Localization of PlNCEDs

**Figure 3:**
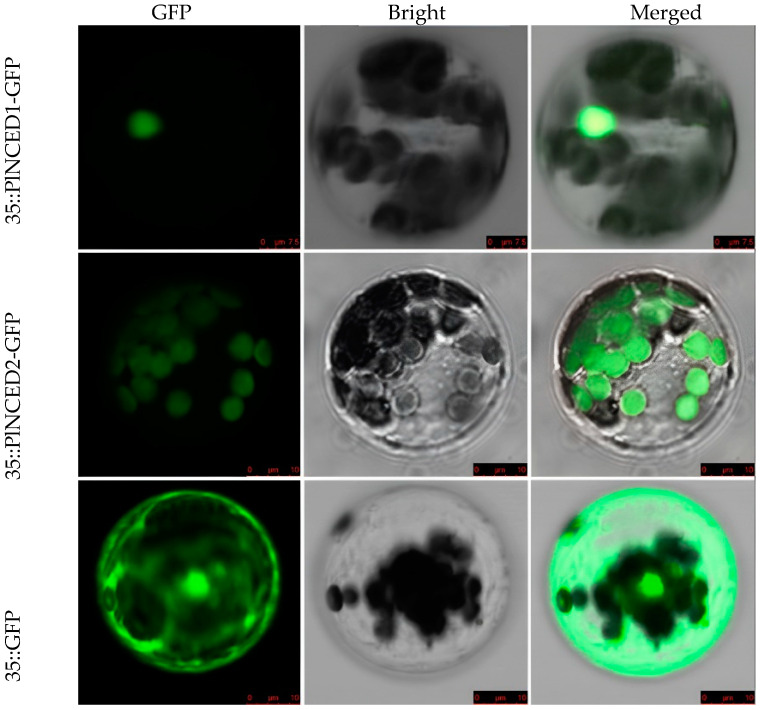
Subcellular localization of the 16318-hGFP-PlNCED fusion protein in protoplast.

Protein maintains its optimal function in a specific subcellular localization. Therefore, to unravel the cellular functions of *PlNCEDs* during seed dormancy and its release process, we also carried out fluorophore tagging of the protein using green fluorescent protein to locate the presence of *PlNCED* proteins within the cell. Fluorescence microscopic analysis showed that the GFP fluorescence signal was distributed in the cell membrane, nucleus, and cytoplasm of *A. thaliana* protoplast containing the empty 16318-hGFP vector (Figure 3). In contrast, the 16318-hGFP-PlNCED1 and 16318-hGFP-PlNCED2 fusion proteins were only observed in the nucleus and cytoplasm, respectively (Figure 3). This differential cellular localization of two PlNCEDs implies that PlNCED1 may play a genetic role similar to that of transcription factors, whereas PlNCED2 may act as a functional enzyme to synthesize ABA.

### 3.4. Functional Analysis of PlNCEDs

**Figure 4:**
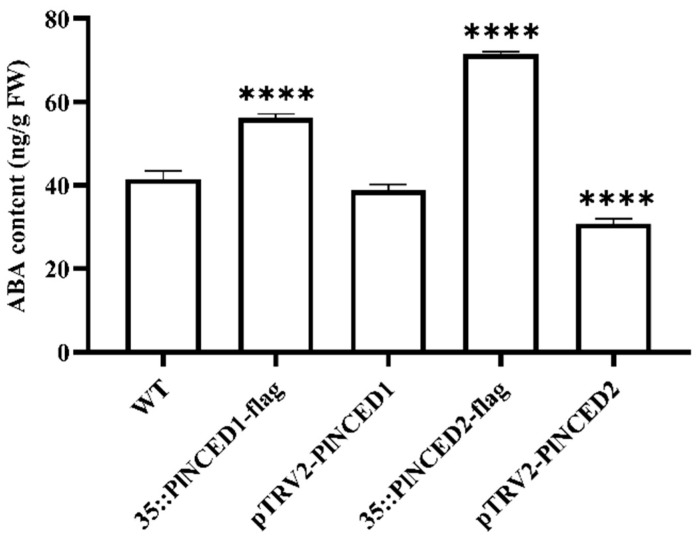
Changes in ABA content in the overexpressed callus of *PlNCEDs*. *PlNCED1* expression in the experimental group (35S::PlNCED1-flag/pTRV2-PlNCED1), *PlNCED2* expression in the experimental group (35S::PlNCED2-flag/pTRV2-PlNCED2), and empty control group (WT). Significant differences (**** *p* ≤ 0.0001) are indicated by asterisks. One-Way ANOVA (*F*-test) analysis was performed using GraphPad Prism 8.0. WT was used as a control.

We first used the transgenic herbaceous peony callus to identify the impact of *PlNCED1* and *PlNCED2* on in vivo ABA content. The results showed that the expression of *PlNCED1* and *PlNCED2* in transgenic herbaceous peony callus was significantly altered compared with the control groups. The expression levels of *PlNCED1* and *PlNCED2* in the overexpressed callus were about 14.5-fold higher than that in the control callus, and the expression levels in the silenced callus were about 0.35-fold lower than that in the control callus, indicating that transgenic calluses were successfully obtained (Figure S6). As shown in Figure 4, the ABA content of the overexpressed *PlNCED* transgenic callus was significantly higher than that of the wild-type callus, while that of the silenced *PlNCED* transgenic callus was much lower, indicating a positive correlation between *PlNCEDs* and endogenous ABA content. In particular, the increase in ABA content caused by *PlNCED2* overexpression was larger than that generated by *PlNCED1* overexpression. We illustrated that *PlNCED2* specifically affects endogenous ABA content by regulating its biosynthesis.

**Figure 5:**
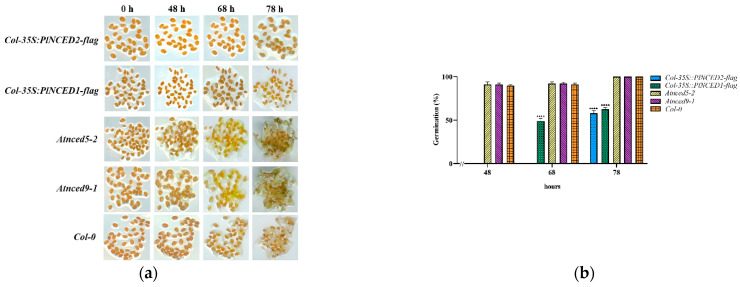

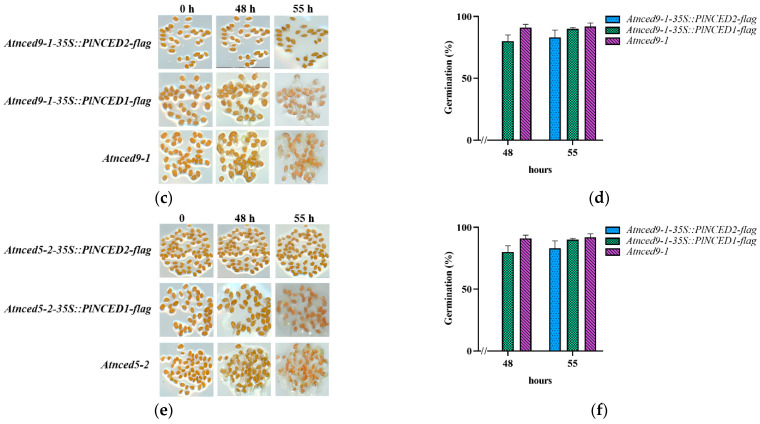
Observation of seed germination rate of different types of *A. thaliana*. (**a,b**) Seed germination rate of overexpressed *PlNCED* transgenic lines (under Col-0 background), Col-0, and mutants (*nced9-1* and *nced5-2*); (**c,d**) seed germination rate of overexpressed *PlNCED* transgenic lines (under *nced9-1* background) and *nced9-1*; (**e,f**) seed germination rate of overexpressed *PlNCED* transgenic lines (under *nced5-2* background) and *nced5-2*. Significant differences (**** *p* ≤ 0.0001) are indicated by asterisks. One-Way ANOVA (*F*-test) analysis was performed using GraphPad Prism 8.0. Col-0 and *Atnced5-2*-*35S::PlNCED2-flag* in (**b**) and (**f**) were used as controls, respectively.

To identify the functional involvement of *PlNCEDs* in seed dormancy, we recorded the seed germination times of *A. thaliana* WT, mutant, transgenic lines, and complementation lines. The germination rates of *A. thaliana* WT and mutant seeds reached about 90% at 48 h, but the seeds of overexpression transgenic lines did not germinate at 48h (Figure 5a,b), indicating that the overexpression of *PlNCEDs* inhibited seed germination. At 68 h, the *PlNCED1* overexpression transgenic line seeds began to germinate, while the *PlNCED2* overexpression transgenic line seeds began to germinate at 78 h (Figure 5a,b), indicating that the inhibitory effects of *PlNCEDs* on seed germination were different, with *PlNCED1* having a slightly weaker impact on seed germination. Compared to *Atnced9-1* and/or *Atnced5-2* mutants, complementation lines induced seed dormancy with a delay and a lower rate of seed germination. In particular, the *PlNCED2* complementation line had a stronger effect (Figure 5c–f). Overall, we demonstrated that *PlNCEDs* inhibited seed dormancy release, and the inhibitory effect of *PlNCED2* was stronger.

## 4. Discussion

*Paeonia lactiflora* is the most familiar herbaceous peony seen in gardens and produces some of the best cut flowers in the floral industry. Though herbaceous peony is one of the most easily grown hardy perennials, its complex double seed dormancy hinders seed germination and consequently imposes adverse effects on breeding and cultivar improvements [24]. Practically, breaking herbaceous peony seed dormancy can be handily achieved through physical (e.g., cold treatments, slitting the seed coat) and biological means (e.g., hormone treatment) [25,26]. The content and level of phytohormones, particularly ABA, are the key factors for natural seed dormancy release. The final concentration of endogenous ABA depends on the dynamic balance between ABA synthesis and catabolism. Therefore, it is critical to know the genes encoding ABA metabolic enzymes and their impacts on herbaceous peony seed dormancy and germination.

To search for genes related to ABA biosynthesis, we identified two *NCED* genes (*PlNCED1* and *PlNCED2*) with differential transcription pre- and post-germination (Figures S4 and S7) based on previously published transcriptome data. Studies have shown that *NCED* genes are the key factors that control the responses of endogenous ABA content to environmental stimuli [37]. The Conserved Domain Database (CDD) search for protein sequences of *PlNCED1* and *PlNCED2* in the NCBI database indicates that *PlNCED1* and *PlNCED2* proteins belong to the RPE65 family, a characteristic conserved domain of enzymes involved in carotenoid cleavage dioxygenase [38,39]. Furthermore, the phylogenetic analysis of *NCED2* clearly revealed its intimate genetic relationship among *P. lactiflora, P. ostii*, and *V. vinifera*. This conclusion suggests that *PlNCED1* and *PlNCED2* have similar functions to *PoNCED and VvNCED*, which play an important rate-limiting role in ABA biosynthesis [40,41].

Previous reports indicate that most NCED proteins are located in chloroplasts [19,42,43]. However, our data show that PlNCED1 is located in the nucleus, but it has no Nuclear Localization Signal (NLS), a short peptide acting as a signal fragment and mediating the transport of proteins from the cytoplasm into the nucleus. Previous studies have shown that not all nucleus-expressed proteins require an NLS, and multiple additional pathways can also mediate their nuclear import [44,45]. One of these pathways is that these proteins without NLSs enter the nucleus by interacting with proteins with NLSs or with other nuclear localization proteins [46]. Therefore, our experiments imply that PlNCED1 may enter the nucleus by relying on the NLSs of other proteins. Additionally, our results suggest that the expression of *PlNCEDs* may be regulated by several miRNAs located in the 3’UTR regions of *PlNCEDs*, as well as *cis*-acting elements located upstream of transcripts (Supplementary Tables S4 and S5). Given the characteristics of these miRNAs, we further demonstrated that *PlNCED1* and *PlNCED2* might play a certain role in seed development and biotic and abiotic stresses, which is consistent with the role of ABA in plant growth. These *cis*-acting elements from the promoter sequences of *PlNCED* genes were presumed to be involved in salicylic acid, ABA, auxin, and jasmonic acid (Supplementary Tables S6 and S7). By combining *PlNCED* genes and their associated transcriptional regulatory binding site predictions, our data provide a preliminary path to explore the molecular mechanisms of ABA and other phytohormones involved in seed dormancy and germination.

The ABA content is proportionally related to the process of herbaceous peony seed dormancy and germination, while ABA accumulation in seeds gradually decreases from dormancy to germination (Figure S5) [36]. By comparing the transcription levels of *PlNCED* genes with ABA contents, we clearly show that the expression dynamics of *PlNCED2* are directly associated with ABA biosynthesis and accumulation after seeds imbibe water (Figures S4 and S5) [36], suggesting that *PlNCED2* is the crucial causal factor for ABA-mediated seed dormancy release in herbaceous peony. The deviation of *PlNCED* expression from ABA content in the imbibition stage may be due to the self-regulation of ABA metabolism genes to adapt to the dynamic balance among endogenous phytohormones after seed imbibition (Figures S4 and S5) [36]. Lee et al. (2018) also found that the level of *NCED1* in orchids (*PtNCED1*) was low in the early stage of seed development but gradually increased and then declined slightly when seeds germinated, and the resulting changes in the seed’s endogenous ABA content played a key role in seed germination [19]. In contrast, the expression level of *NCED* in habanero pepper was high during seed germination, but the content of ABA gradually decreased during the same time span. This indicates that *NCED* has less of an effect on ABA synthesis and a weaker impact on seed germination in habanero pepper [47]. All of these reports indicate that *NCED* has different regulatory effects on ABA content in different plant species. Lastly, our transgenic experiment in herbaceous peony has also proved that *PlNCED2* plays a major role in ABA biosynthesis and accumulation and subsequently contributes to seed dormancy maintenance.

The ultimate way to dissect the biological function of *PlNCED* genes involved in seed dormancy is to generate transgenic lines of herbaceous peony. However, it is difficult to establish a stable and efficient genetic transformation system for herbaceous peony [48]. Although the genetic transformation system of herbaceous peony has been used for the functional analysis of some genes in recent years [49,50,51], it takes at least 3 years to obtain transgenic seeds from transgenic seedlings due to the growth characteristics of herbaceous peony. In order to efficiently verify the effect of *PlNCED* genes on the seed germination rate, we used *A. thaliana*, a typical short-growth-cycle model plant, for subsequent gene transformation experiments in this study [52]. *Arabidopsis thaliana* mutants play an important role in revealing the growth and development of different plants. *AtNCED5* and *AtNCED9* mutants in *A. thaliana* have been found to affect ABA production in the embryo and endosperm, leading to seed dormancy [15,16]. Therefore, we built *PlNCED* transgenic lines in wild-type *A. thaliana* as well as *PlNCED* complementation lines in *A. thaliana NCED* mutants to explore the role of *PlNCED* in seed dormancy and germination. Phenotyping *PlNCED* transgenic lines indeed showed that weak seed dormancy was induced to a certain extent by the overexpression of *PlNCEDs* in *A. thaliana* (Figure 5a,b). More significantly, the seed germination rate of the *PlNCED2* complementation line was significantly lower than that of the *nced5-2* mutant (Figure 5f), indicating that overexpressed *PlNCED2* rescued the partial function of *Arabidopsis NCED* genes. Collectively, the biological function of *PlNCEDs* is consistent to that of *A. thaliana NCEDs* by promoting seed dormancy and delaying germination.

## 5. Conclusions

In summary, we identified and cloned two genes from the *NCED* gene family, which are related to ABA synthesis in herbaceous peony. By comparing their protein sequences and phylogenetics with those of homologs in other plant species, we were able to detect their functional conserved domain. The dynamics of *PlNCED2* transcription epistatically regulated endogenous ABA biosynthesis and accumulation. Using transgenic and complementation rescue lines in *Arabidopsis*, we were able to demonstrate the phenotypic traits of *PlNCED* genes, which induce seed dormancy and hinder seed germination. Our data and analysis provide the first step to understanding the underlying molecular and genetic mechanisms of complex double seed dormancy in herbaceous peony.

## Data Availability

The datasets generated and analyzed during the current study are available in the NCBI repository—*PlNCED1*: OL744236, https://www.ncbi.nlm.nih.gov/nuccore/OL744236, *PlNCED2*: OL744237, https://www.ncbi.nlm.nih.gov/nuccore/OL744237.

## Supplementary Materials

The following supporting information can be downloaded at https://www.mdpi.com/article/10.3390/plants12040710/s1. Figure S1: Identification of *nced* homozygous mutants in *A. thaliana*.; Figure S2: Nucleotide and amino acid sequences of the *PlNCED* gene coding regions; Figure S3: Conserved domain prediction for the proteins encoded by *PlNCEDs*; Figure S4: *PlNCED* expression at different stages of seed dormancy release in herbaceous peony; Figure S5: Changes in ABA content at different seed dormancy release stages in herbaceous peony; Figure S6: Identification of *PlNCED* transgenic callus; Figure S7: Other *PlNCED* family members’ expression at different stages of seed dormancy release in herbaceous peony; Table S1: Primers used in this study; Table S2: The identification of PlNCED1 from different plant species (%); Table S3: The identification of PlNCED1 from different plant species (%); Table S4: Prediction of miRNA targets in 3’UTR of *PlNCED1*; Table S5: Prediction of miRNA targets in 3’UTR of *PlNCED2*; Table S6: Prediction of *cis*-acting elements in promoter sequence of *PlNCED1*; Table S7: Prediction of *cis*-acting elements in promoter sequence of *PlNCED2*.
